# Insights into heterosis from histone modifications in the flag leaf of inter-subspecific hybrid rice

**DOI:** 10.1186/s12870-024-05487-6

**Published:** 2024-08-12

**Authors:** Tianpu Qi, Mengyao Wang, Peixuan Wang, Linyou Wang, Jianbo Wang

**Affiliations:** 1 State Key Laboratory of Hybrid Rice, College of Life Sciences, Wuhan University, Wuhan, 430072 China; 2 Institute of Crop and Nuclear Technology Utilization, Zhejiang Academy of Agricultural Sciences, Hangzhou, 310021 China

**Keywords:** Inter-subspecific hybrid rice, Heterosis, Histone modification, DNA methylation, Gene expression

## Abstract

**Background:**

Inter-subspecific hybrid rice represents a significant breakthrough in agricultural genetics, offering higher yields and better resilience to various environmental stresses. While the utilization of these hybrids has shed light on the genetic processes underlying hybridization, understanding the molecular mechanisms driving heterosis remains a complex and ongoing challenge. Here, chromatin immunoprecipitation-sequencing (ChIP-seq) was used to analyze genome-wide profiles of H3K4me3 and H3K27me3 modifications in the inter-subspecific hybrid rice ZY19 and its parents, Z04A and ZHF1015, then combined them with the transcriptome and DNA methylation data to uncover the effects of histone modifications on gene expression and the contribution of epigenetic modifications to heterosis.

**Results:**

In the hybrid, there were 8,126 and 1,610 different peaks for H3K4me3 and H3K27me3 modifications when compared to its parents, respectively, with the majority of them originating from the parental lines. The different modifications between the hybrid and its parents were more frequently observed as higher levels in the hybrid than in the parents. In ZY19, there were 476 and 84 allele-specific genes with H3K4me3 and H3K27me3 modifications identified, representing 7.9% and 12% of the total analyzed genes, respectively. Only a small portion of genes that showed differences in parental H3K4me3 and H3K27me3 modifications which demonstrated allele-specific histone modifications (ASHM) in the hybrid. The H3K4me3 modification level in the hybrid was significantly lower compared to the parents. In the hybrid, DNA methylation occurs more frequently among histone modification target genes. Additionally, over 62.58% of differentially expressed genes (DEGs) were affected by epigenetic variations. Notably, there was a strong correlation observed between variations in H3K4me3 modifications and gene expression levels in the hybrid and its parents.

**Conclusion:**

These findings highlight the substantial impact of histone modifications and DNA methylation on gene expression during hybridization. Epigenetic variations play a crucial role in controlling the differential expression of genes, with potential implications for heterosis.

**Supplementary Information:**

The online version contains supplementary material available at 10.1186/s12870-024-05487-6.

## Introduction

Heterosis, commonly referred to as hybrid vigor, refers to the phenomenon that hybrid offspring exhibit superior traits compared to their parents. These enhanced traits encompass various aspects such as growth, yield, biomass, stress tolerance, and resistance to diseases [1, 2]. Quantitative genetics theories for heterosis involve dominance, overdominance, and epistasis hypotheses [3, 4]. Presently, advancements in molecular detection and quantification technologies have enabled the confirmation of these hypotheses at the molecular level for diverse traits across various species [5, 6]. Recent research has employed cutting-edge tools such as genome-wide and transcriptome-wide association studies [7, 8], long-read sequencing [2], and three-dimensional (3D) genome scanning [9] to identify numerous genetic loci crucial for genomics-driven hybrid breeding. Additionally, gene regulatory network analysis [10] and single-cell transcriptome profiling [11] have identified critical gene expression alterations underlying heterosis. Despite substantial research and its extensive application, the molecular mechanisms underlying heterosis remain elusive.

Asian cultivated rice (*Oryza sativa* L.) encompasses two primary subspecies: *indica* (*O. sativa* L. subsp. *indica* Kato) and *japonica* (*O. sativa* L. subsp. *japonica* Kato), and these two subspecies possess genomes that are closely related, with high levels of homology and synteny [12]. Recent studies have shown that hybrids resulting from crosses between *indica* and *japonica* subspecies exhibit greater heterosis than those resulting from crosses within subspecies, which is of potential production and research value [13, 14]. The heterosis has been widely used in hybrid rice and resulted in significant achievements in grain yield. Inter-subspecific hybrid rice yields are often 10–20% higher than corresponding inbred rice cultivars [15, 16].

The flag leaves of rice play a crucial role during growth and development. They serve as the primary photosynthetic organs during the filling stage, providing essential energy and carbohydrates for grain development and maturation [17]. Moreover, flag leaves are vital for nutrient and assimilate transfer to grains, ensuring sufficient nutrition [18]. Additionally, flag leaves exhibit notable contributions to heterosis, primarily enhancing growth and yield in hybrid rice through gene expression and epigenetic regulatory mechanisms [19]. Research has highlighted the significant role of non-coding RNAs in regulating flag leaf gene expression and heterosis [20]. However, compared to other yield-related factors, research on flag leaves remains relatively scarce.

Histone octamers consist of 146 bp DNA-wrapped four core histones (two copies each of histones H2A, H2B, H3, and H4). Post-translational modifications of histones, such as methylation, acetylation, phosphorylation, and ubiquitination, may be epigenomic factors that regulate genomic activity and gene expression [21–23]. H3K4me3 (Histone H3 Lysine 4 trimethylation) is typically regarded as an active chromatin mark. It is predominantly located near the core promoters of transcription start sites, facilitating the binding of RNA polymerase and gene transcription. H3K27me3 (Histone H3 Lysine 27 trimethylation) serves as a repressive chromatin mark, and it is often found in transcriptionally silenced regions such as the promoters and enhancers of silent genes, playing a critical role in gene silencing and epigenetic regulation. Together, they modulate chromatin states, crucially influencing the timing and level of gene expression, and impacting cellular functions and developmental processes. Gene expression is regulated by the combination of genetic and epigenetic mechanisms that determine the growth and development of plants and animals. Epigenetic mechanisms involve DNA methylation and histone modifications and others, which play crucial roles in regulating gene expression and maintaining cellular identity.

An important aspect of epigenetic studies is the inheritance and variation of DNA or histone modifications in hybrid genomes containing newly merged distinct subgenomes. Epigenomic studies have demonstrated that variation in DNA methylation and histone modifications between hybrids and parents were associated with altered gene expression patterns in hybrids, which affect gene activity changes in hybrids and contribute to heterosis [24, 25]. Indeed, many studies on DNA methylation have been accumulated [19, 26–28], while much less research has been conducted on histone modifications in hybrids [29–32].

In this study, H3K4me3 and H3K27me3 modifications were analyzed by chromatin immunoprecipitation followed by deep sequencing (ChIP-seq) approach in the flag leaf of the inter-subspecific hybrid rice ZY19, and its parents Z04A and ZHF1015. The effects of histone modifications on gene expression were investigated, together with DNA methylation, to explore the role of epigenetic modifications on gene expression. The effects of differential histone modifications between parents on allele-specific histone modifications (ASHM) in hybrid were analyzed, intending to find evidence for the emergence of heterosis. Overall, the mechanism of heterosis was revealed at the level of transcriptional and epigenetic modifications, and abundant data for future hybrid breeding was provided.

## Materials and methods

### Plant materials and growth conditions

Rice cultivars Z04A (*japonica*, maternal line) and ZHF1015 (*indica*, paternal line) and their hybrid ZY19 [19, 33] were used in this study. Seedlings were grown in the experimental field of Wuhan University, Wuhan, China. ZY19 and its parents were grown using conventional field management methods. The flag leaves were sampled from the hybrid and its parents at the heading stage, frozen immediately in liquid nitrogen for 5–6 h, and stored at -80 °C until use.

### ChIP-seq library construction and sequencing

Chromatin immunoprecipitation (ChIP) was performed on three genotypes according to the established protocol [34]. The antibodies used were H3K4me3 (ab8580; Abcam, Cambridge, UK) and H3K27me3 (07-449; Sigma-Aldrich, Darmstadt, Germany). The high-throughput DNA sequencing libraries corresponding to 200–500 bps were prepared by using VAHTS Universal DNA Library Prep Kit for Illumina V3 (Catalog NO. ND607, Vazyme), and enriched, quantified, and finally sequenced on the DNBSEQ-T7 sequencer (MGI Tech Co. Ltd. China) with PE150 model.

### ChIP-seq data analysis

Quality control of the sequencing data was performed. Raw sequencing data was first filtered by Trimmomatic (version 0.36) [35], and low-quality reads were discarded while the reads contaminated with adaptor sequences were trimmed. The clean reads were used for protein binding site analysis. The clean data were aligned with the rice reference genome (MSU7.0 (http://rice.plantbiology.msu.edu/)) using STAR software (version 2.5.3a) with default parameters. The reads distribution was analyzed using RseQC (version 2.6) and the peaks were called using Macs2 (Version 2.1.1). The bedtools (Version 2.25.0) were used for peak annotation and peak distribution analysis. In the histone ChIP-seq project with Input and IP, we used Input as the background and Epic2 to call peak the IP.

### Differential histone modification peaks identification

The differential histone-modified peaks were analyzed with CSAW in edgeR, using Fisher’s exact test. Each peak adjusted by fold change (|log_2_(FC)| ≥ 1) and *P*-value (*P* < 0.01) was defined as a differential histone modification (DHM) peak.

### RNA-seq library preparation and sequencing

Total RNA was extracted from three replicates of various samples using Trizol Reagent (15596026CN, Invitrogen, Carlsbad, CA, USA). KCTM Stranded mRNA Library Prep Kit for Illumina^®^ (Catalog NO. DR08402, Wuhan Seqhealth Co. Ltd. China) was used for RNA sequencing library preparation by 2 µg total RNAs following the manufacturer’s instruction. PCR products corresponding to 200–500 bps were enriched, quantified, and finally sequenced on DNBSEQ-T7 sequencer (MGI Tech Co., Ltd. China) with PE150 model.

### RNA-seq data analysis

Raw sequencing data was filtered by Trimmomatic (version 0.36) [35]. Clean data were mapped to the reference MSU7.0 rice genome (http://rice.plantbiology.msu.edu/) using STAR software (version 2.5.3a) with default parameters. The gene expression of each sample was counted by featureCounts (Subread-1.5.1; Bioconductor), and then RPKMs (reads per kilobase genic region per million mapped reads) were calculated [36].

The differentially expressed genes (DEG) were identified using the edgeR package (version 3.12.1) with FDR (FDR < 0.05) and fold change (|log_2_FC| ≥ 1). Gene ontology (GO) analysis and Kyoto Encyclopedia of Genes and Genomes (KEGG) enrichment analysis for genes were both conducted by KOBAS software (version 2.1.1) with a *P*-value ≤ 0.05.

### Identification of SNPs and allele-specific expression (ASE) detection

SNPs were collected from RNA-seq reads in each sample using GATK (version 4.1.9) [37]. Maternal or paternal-specific SNPs were screened for consistency across three biological replicates, retaining loci with read counts ≥ 1 in all accessions and replicates. For allele-specific expression (ASE) analysis, the hybrid ZY19 reads were categorized based on maternal or paternal-specific SNPs, and the maternal and paternal allele counts were obtained for each gene. ASE was quantified using ASEReadCounter, with a threshold of |log_2_(M/P)| ≥ 1 and *P* < 0.05, followed by bias determination with the GeneiASE program using Student’s t-test. Finally, allele-specific expression genes (ASEGs) with the same directional bias were identified in the F1 hybrid.

### Determination of allele-specific histone modification(ASHM)

The allele-specific histone modification (ASHM) in the ZY19 was distinguished based on SNPs. The filtered SNP list obtained from the mRNA of histone modification target genes was used to count the SNPs in the hybrid ChIP-seq bam files. For a given SNP site, if the paternal line is homozygous for the mutation and the maternal line is homozygous for the non-mutation, then reads matching the reference genome at this site in the hybrid are from the maternal line, while mutated reads are from the paternal line. Conversely, if the maternal line is homozygous for the mutation and the paternal line is homozygous for the non-mutation, then mutated reads are from the maternal line, and reads matching the reference genome are from the paternal line. The ASHM genes were determined based on the reads in the gene body region [38]. Histone modifications of alleles were classified into three categories [29].

### DNA methylation data analysis

The process of constructing Whole‑genome bisulfite sequencing (WGBS) libraries can be found in our previous study [19]. With default parameters, the clean data eliminated were aligned to the reference genome of rice (MSU_v7.0) (http://rice.plantbiology.msu.edu/). Methylation sites were anticipated utilizing the Bismark mutation extractor [39], and the percentage of clean reads was computed.

### Assessment of methylated cytosine (C site)

Methylation levels were evaluated for the C site in accordance with Schultz et al. [40]. Schultz et al. employ Whole Genome Bisulfite Sequencing (WGBS) to assess the methylation status of individual cytosine (C) sites. They utilize sodium bisulfite treatment to convert unmethylated cytosines to uracil and subsequently thymine via PCR, followed by sequencing and alignment to a reference genome. This method distinguishes methylated (C remains) from unmethylated (C converted to T) cytosines across a population of cells, providing a site-specific methylation level calculated as the ratio of methylated reads to total reads covering each site. Analysis typically includes combining read counts from both DNA strands for CG sites and applying a binomial test to assess methylation frequencies above background noise.

### Identification of differentially methylated regions (DMRs)

Differentially methylated regions (DMRs) were scrutinized using MOABS [41]. Identification of differentially methylated regions (DMRs) involves analyzing genomic regions where there are significant differences in methylation levels between samples or conditions. Typically, DMRs are defined as contiguous genomic regions with at least three differentially methylated sites, exhibiting a methylation level difference exceeding a specified threshold (e.g., 0.4 for most contexts, 0.2 for specific contexts like CHG and CHH), and achieving statistical significance (often assessed by Fisher’s exact test with a *P*-value < 0.05). This approach ensures robust detection of regions where DNA methylation patterns vary significantly.

### The statistical tests

Statistical significance for all comparisons in this study was assessed using R (version 4.0.3) (R Foundation for Statistical Computing, Vienna, Austria; https://www.r-project.org). Various statistical tests were utilized, such as the exact binomial test, Chi-squared test, Kolmogorov–Smirnov test (K-S test), Student’s *t*-test, Wilcoxon rank sum test, and Pearson’s product-moment correlation.

## Results

### Genome-wide profiles of histone modifications between the hybrid and its parents

**Fig. 1:**
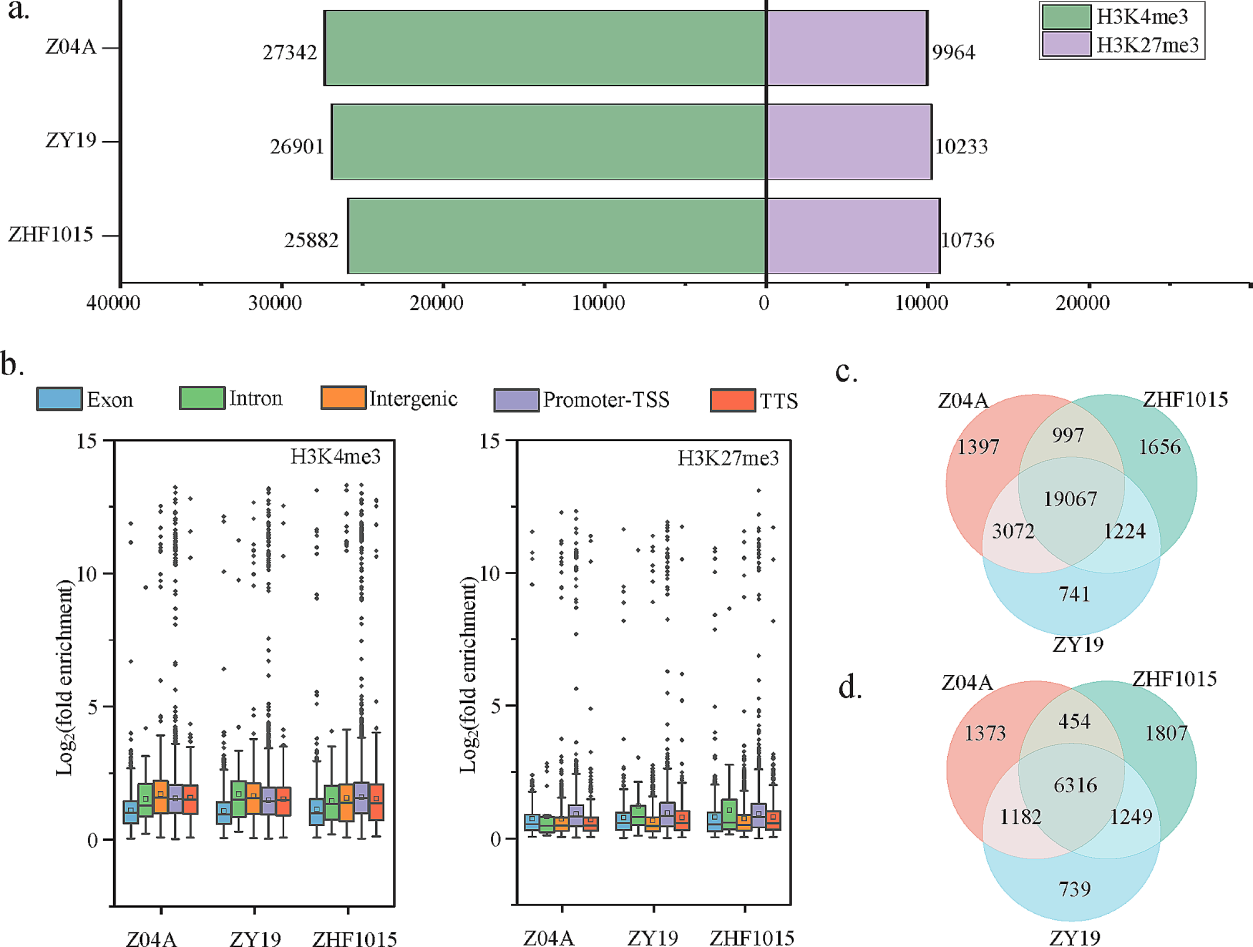
Histone modification profiles of hybrid and its parents. (**a**) Peaks number of the hybrid and its parents. (**b**) Level of histone modification in different regions of the three cultivars. (**c-d**) Venn diagrams of peak annotated genes in H3K4me3 (**c**) and H3K27me3 (**d**)

Rice cultivars Z04A and ZHF1015 represent the two subspecies (*japonica* and *indica*) of *O. sativa* L., and their resultant F1 ZY19 is an inter-subspecific hybrid. For the inquiry into histone modification status in the flag leaf tissues of the three cultivars, genome-wide levels of H3K4me3 and H3K27me3 modifications were analyzed. Chromatin immunoprecipitation was performed and 12 libraries were generated using flag leaf tissue at the heading stage (Table S1). In the histone ChIP-seq study employing Input and IP, Input was utilized as the baseline for peak calling in the IP dataset. Among the three cultivars, the number of peaks enriched with H3K4me3 modification was highest in the maternal line, while H3K27me3 modification was highest in the paternal line. In the hybrid, the number of peaks enriched with both histone modifications was intermediate (Fig. 1a; Table S2). For the chromosomal level analyses, the frequency of modification peaks was lower on Chr9 and Chr10 compared to Chr1 (Fig. S1a, b). Across most chromosomes, the abundance of peaks enriched with H3K4me3 modification was highest in the maternal line and lowest in the paternal line, in contrast to H3K27me3 (Fig. S1a, b). The 2 kb upstream and downstream region of the transcription start site, annotated as the promoter region, was referred to as the promoter-TSS. The centers of peaks enriched with both histone modifications were primarily located at the promoter-TSS (Fig. S1c). The enrichment level of histone modifications was described by fold enrichment. Generally, the fold enrichment of H3K4me3 peaks was higher overall compared to those of H3K27me3, and their fold enrichment in exon regions was significantly lower than in other regions. H3K27me3 peaks exhibit higher fold enrichments in the promoter-TSS (Fig. 1b).

Annotating peaks to genes, the number of genes targeted by H3K4me3 were 24,529, 24,104, and 22,940 in Z04A, ZY19, and ZHF1015, respectively. For H3K27me3, the numbers were 9,325, 9,486, and 9,826 in the three lines respectively. Most genes had only 1 peak on their sequence, while a few genes had multiple peaks, with a maximum of 7 peaks on a gene. Longer genes often have more histone modification peaks due to their more complex regulatory regions and structures. There was a notable discrepancy between the abundance of H3K4me3 target genes, with the maternal line displaying the highest count and the paternal line the lowest count, which contrasted with H3K27me3 target genes (Table S3). The number of peaks and genes in both modifications of the hybrid was between its parental lines, which may be related to hybridization (Fig. 1a). The Venn diagram analysis of H3K4me3 target genes revealed a total of 19,067 genes across the three lines. Among these, there were 3,072 genes common to both maternal and hybrid lines, 1,224 genes common to paternal and hybrid lines, and only 741 genes exclusive to the hybrid line (Fig. 1c). Similarly, the analysis of H3K27me3 target genes showed 6,316 genes across the three lines, with 1,182 genes common to maternal and hybrid lines, 1,249 genes common to paternal and hybrid lines, and 739 genes exclusived to the hybrid alone (Fig. 1d). In terms of overlapping target genes, the hybrid line exhibited a greater overlap with the maternal line H3K4me3 target genes, whereas it showed a greater overlap with the paternal line H3K27me3 target genes. It is noteworthy that a significant proportion of H3K27me3 target genes were exclusive to the hybrid line (Fig. 1d).

### The differential histone modification regions between the hybrid and its parents

**Fig. 2:**
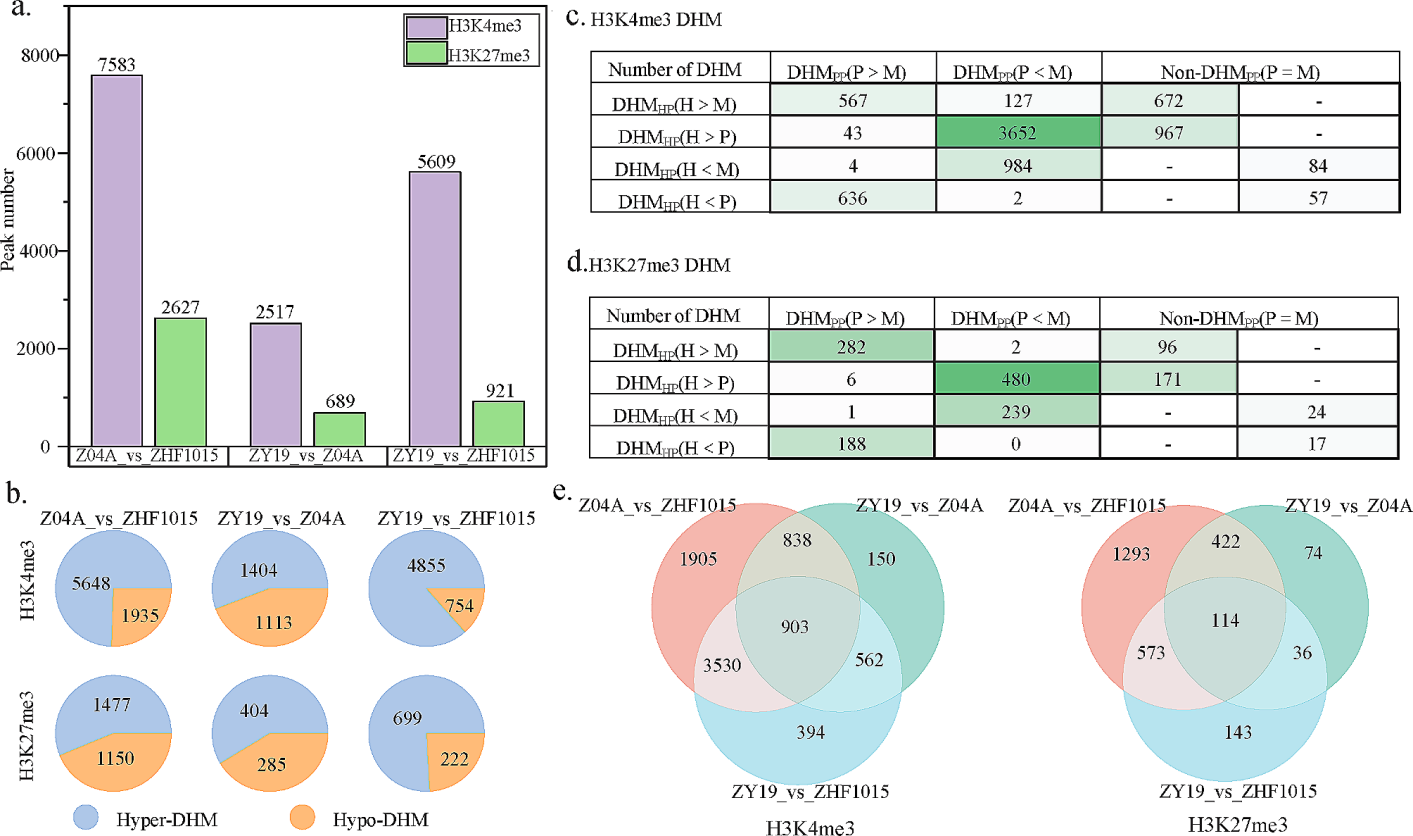
Differential histone modification profiles of the hybrid and its parents. (**a**) Number of differential peaks for H3K4me3 and H3K27me3 modification. (**b**) Number of hyper- and hypo-DHMs in the hybrid and its parents. (**c-d**) Histone modification patterns of DHMs between the hybrid and its parents. H, M, and P denote the hybrid ZY19, the maternal line Z04A, and the paternal line ZHF1015, respectively. (**e**) Venn diagrams of DHM genes in the hybrid and its parents

There were 7,583 and 2,627 differential peaks for H3K4me3 and H3K27me3 modifications respectively between the parents. In the hybrid, there were 8,126 and 1,610 different peaks for H3K4me3 and H3K27me3 modifications when compared to its parents, respectively, with the majority of them originating from the parental lines. The differential histone modifications between the hybrid and its parents were much less than those between parental lines (Fig. 2a; Table S4). The number of differential modifications between the hybrid and maternal line was fewer than that with the paternal line in both histone modifications. This suggested that the hybrid was more similar to the maternal line at the level of histone modifications. Differential histone modifications (DHM) were categorized into hyper-DHM and hypo-DHM, based on different orientations. Hyper-DHM and hypo-DHM were defined as differential histone modifications characterized by increased and decreased levels of specific histone marks, respectively, in comparisons between two cultivars. The proportion of hyper-DHMs was greater in the hybrid than in hypo-DHMs (Fig. 2b). The following analyzed the histone modification patterns between the hybrid and its parents. After excluding DHMs in different directions, DHMs between parents were defined as DHM_PP_s, while DHMs between hybrid and its parents were referred to as DHM_HP_s. H, M, and P denoted the hybrid ZY19, the maternal line Z04A, and the paternal line ZHF1015, respectively. Through these definitions, the differences in histone modification levels between the hybrid and its parents could be clearly distinguished. For example, DHM_PP_(P > M) indicated that the histone modification level in the paternal line ZHF1015 was higher than in the maternal line Z04A, while DHM_PP_(P < M) indicated that it was lower. Similarly, DHM_HP_(H > M or P) indicated that the histone modification level in the hybrid ZY19 was higher than in the maternal or paternal line, whereas DHMHP (H < M or P) indicated that it was lower. In terms of H3K4me3, 567 (41.51%) of the DHM_HP_(H > M) originated from DHM_PP_(P > M) genes. Furthermore, among the DHM_HP_(H < M) genes, 984 (91.79%) were traced back to DHM_PP_(P < M) (Fig. 2c). For the hybrid and paternal line, there were 3,652 (78.34%) DHM_HP_(H > P) and 636 (91.51%) DHM_HP_(H < P) genes originated from DHM_PP_, respectively (Fig. 2c). Notably, the ratio of DHM_HP_(H > M or P) originated from DHM_PP_ genes (41.51% and 78.34%) was considerably lower compared to that of DHM_HP_(H < M or P)s (91.79% and 91.51%). Moreover, there were several DHM_HP_(H > M or P) genes (49.19% and 20.74%) exhibiting no histone modification disparities between parents. A similar pattern was found in the H3K27me3 modification (Fig. 2d). This suggested that hybrid generated a greater number of histone modifications at higher levels compared to its parents, potentially aiding in the regulation of gene expression.

The overlapping analysis of H3K4me3_DHM genes reveals that 562 genes, exhibiting differences between the hybrid and both parents, were not derived from DHM_PP_s, accounting for 6.8% of all H3K4me3_DHM genes. These genes exhibited distinctions from their parental modifications, potentially playing a role in heterosis manifestation. Additionally, 150 H3K4me3_DHM genes displayed differences exclusively between the hybrid and the maternal line, while 394 genes exhibited discrepancies solely between the hybrid and the paternal line (Fig. 2e). In the context of H3K27me3_DHM, 36 genes demonstrated distinctions between the hybrid and both parental lines, whereas 74 genes exhibited variances solely between the hybrid and the maternal line and 143 genes displayed differences exclusively between the hybrid and the paternal line (Fig. 2e). After enrichment analysis of Gene Ontology (GO) annotation, H3K4me3_DHM genes were enriched to a total of 119 entries between parents; 99 entries between the hybrid and the maternal line; and 116 entries between the hybrid and the paternal line. H3K27me3_DHM genes were enriched to 129, 47, and 77 entries between parents, the hybrid and the maternal line, and the hybrid and the paternal line, respectively (Table S5). Here, the top 20 GO terms, sorted by *P*-value, were selected for plotting (Fig. S2). Due to the fact that DHM_PP_s primarily originated from DHM_HP_s, GO terms not enriched between parents were of particular interest. Between the hybrid and the maternal line, “nucleotide binding, GO:0000166”, “calcium ion binding, GO:0005509”, “negative regulation of DNA recombination, GO:0045910”, “nucleosomal DNA binding, GO:0031492”, and “chromosome condensation, GO:0030261” were not enriched between parents. Between the hybrid and the paternal line, “embryo development ending in seed dormancy, GO:0009793” and “amine metabolic process, GO:0009308” were not enriched between parents. For H3K27me3, “defense response to fungus, incompatible interaction, GO:0009817” was not enriched between parents in the hybrid and the maternal line, and the top 20 GO terms were all enriched between parents in the hybrid and the paternal line.

### Inheritance and remodeling of allele-level histone modifications in the hybrid

**Fig. 3:**
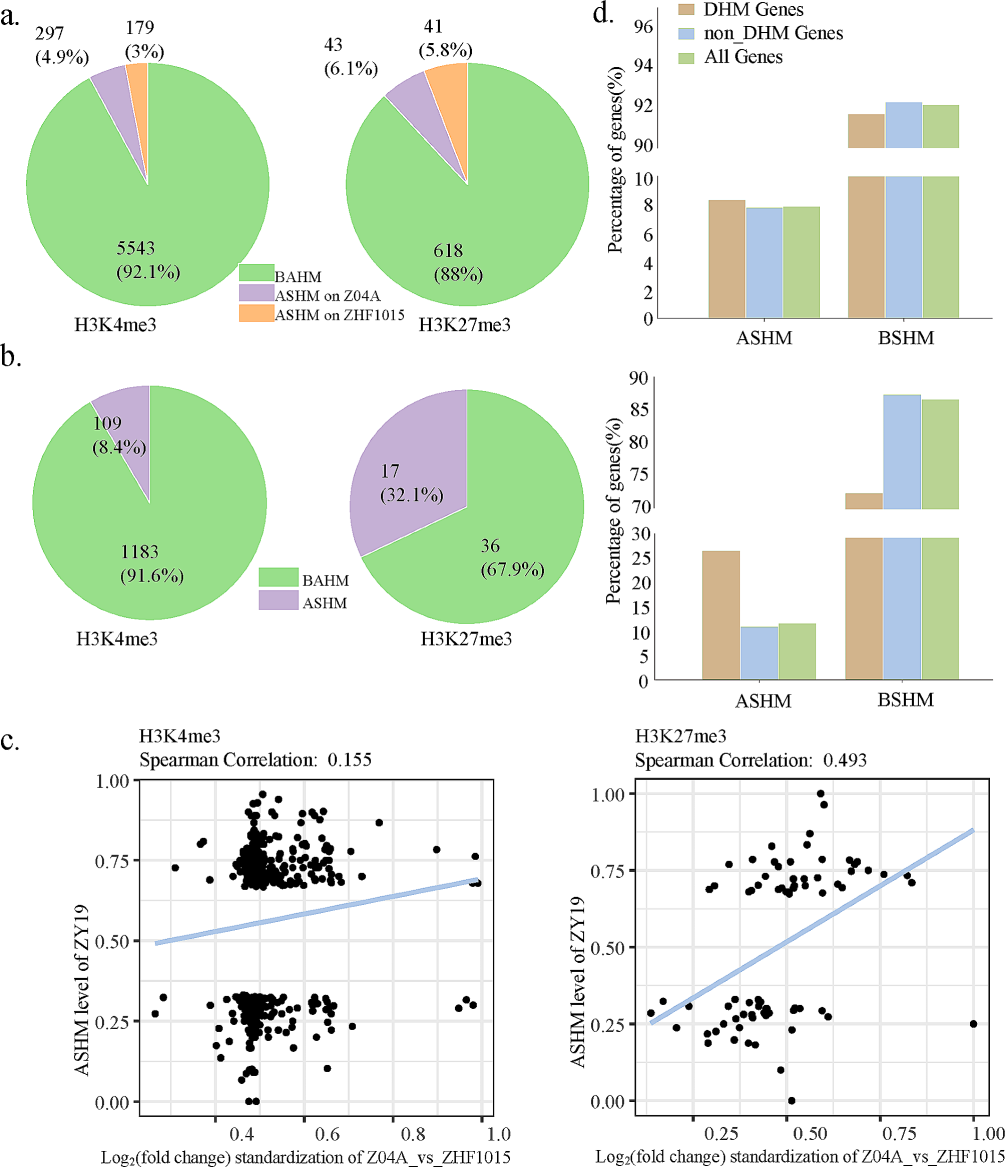
Allele-level histone modifications in the hybrid. (**a**) Allele-level H3K4me3 and H3K27me3 modification types. (**b**) Allele-level histone modifications of DHM_PP_ genes in the hybrid. (**c**) The correlation between the level of DHM_PP_ and ASHM in the hybrid. (**d**) DHM_HP_ genes of Allele-level histone modifications

Genes targeted by histone modification and possessing more than 9 reads containing SNP sites were screened within the CDS region. In the hybrid, 6,019 and 702 genes were available for H3K4me3 and H3K27me3 modification analysis of alleles. The allele-level histone modifications that were derived from the paternal or maternal alleles were calculated by dividing the reads of each allele by the total number of reads. For a gene of the hybrid, the ratio (0 to 1) of the number of SNP-reads detected from the maternal line genotype to the total number of them was utilized as a value to quantify allele-level histone modification (Table S6) [29]. Genes with the value in the range of 1/3 to 2/3, indicating that the level of histone modification differences between alleles was less than 2-fold, were defined as biallelic histone modifications (BAHM). Genes with the value outside the range of 1/3 to 2/3 were defined as allele-specific histone modifications (ASHM). ASHM genes can be further classified into two categories according to the parental alleles with higher modification levels. Genes with the value to quantify allele-level histone modification less than 1/3 were defined as ASHM on ZHF1015; conversely, genes with the value higher than 2/3 were defined as ASHM on Z04A. In ZY19, there were 476 and 84 allele-specific genes with H3K4me3 and H3K27me3 modifications identified, representing 7.9% and 12% of the total analyzed genes, respectively. Among the allele-level histone modifications in ZY19, there were 297 ASHM on Z04A and 179 ASHM on ZHF1015 for H3K4me3, while 43 on Z04A and 41 on ZHF1015 for H3K27me3, respectively (Fig. 3a).

The analysis examined the association between DHM_PP_s and variations in allele modifications in the hybrid. Of the allele-level H3K4me3 genes, 1,292 were identified as H3K4me3_DHM_PP_s. Among these, 1,183 genes (91.6%) exhibited BAHM, while 109 genes (8.4%) demonstrated ASHM. Regarding H3K27me3, a total of 53 genes were classified as H3K27me3_DHM_PP_s. Of these, 36 genes (67.9%) exhibited BAHM, whereas 17 genes (32.1%) demonstrated ASHM (Fig. 3b). These findings indicated that despite a subset of genes retaining allelic-level modification differences between parental lines in hybrid, histone modification remodeling occurred in the majority of genes. Among the genes examined, *GDP-l-galactose phosphorylase* (*OsGGP*, LOC_Os12g08810), a critical enzyme implicated in spike development and photosynthesis regulation, was highlighted as an example. While *OsGGP* displayed a significant difference in parental H3K4me3 modification, it underwent allele-specific histone modification remodeling in the hybrid and eliminated the parental distinction. The correlation coefficients for H3K4me3 and H3K27me3 between the level of histone modifications difference in the parents and ASHM in the hybrid were determined to be Spearman correlation = 0.155 and Spearman correlation = 0.493, respectively (Fig. 3c). In the hybrid, allele-level modification exhibited a weak correlation with parental differences in H3K4me3 and moderate correlation in H3K27me3 (Fig. 3c). The DHM_PP_ genes comprised DHM_HP_ genes to a certain extent, and differences in histone modifications between parents were correlated with ASHM in hybrid. Consequently, ASHM contributed to a higher proportion of DHM_HP_ genes. For H3K27me3, ASHM was notably enriched in DHM_HP_ genes compared to the genomic average, consistent with previous findings indicating a modest association between histone modification differences and ASHM (Fig. 3d).

Allele-specific expression (ASE) was regarded as a mechanism of heterosis [42]. Genes exhibiting both ASE and ASHM bias were identified through screening (Table S6). Remarkably, congruent findings were observed for both modifications: a greater number of genes displayed ASE bias consistent with ASHM (Fig. S3b).

### The impact of histone modifications on gene expression

**Fig. 4:**
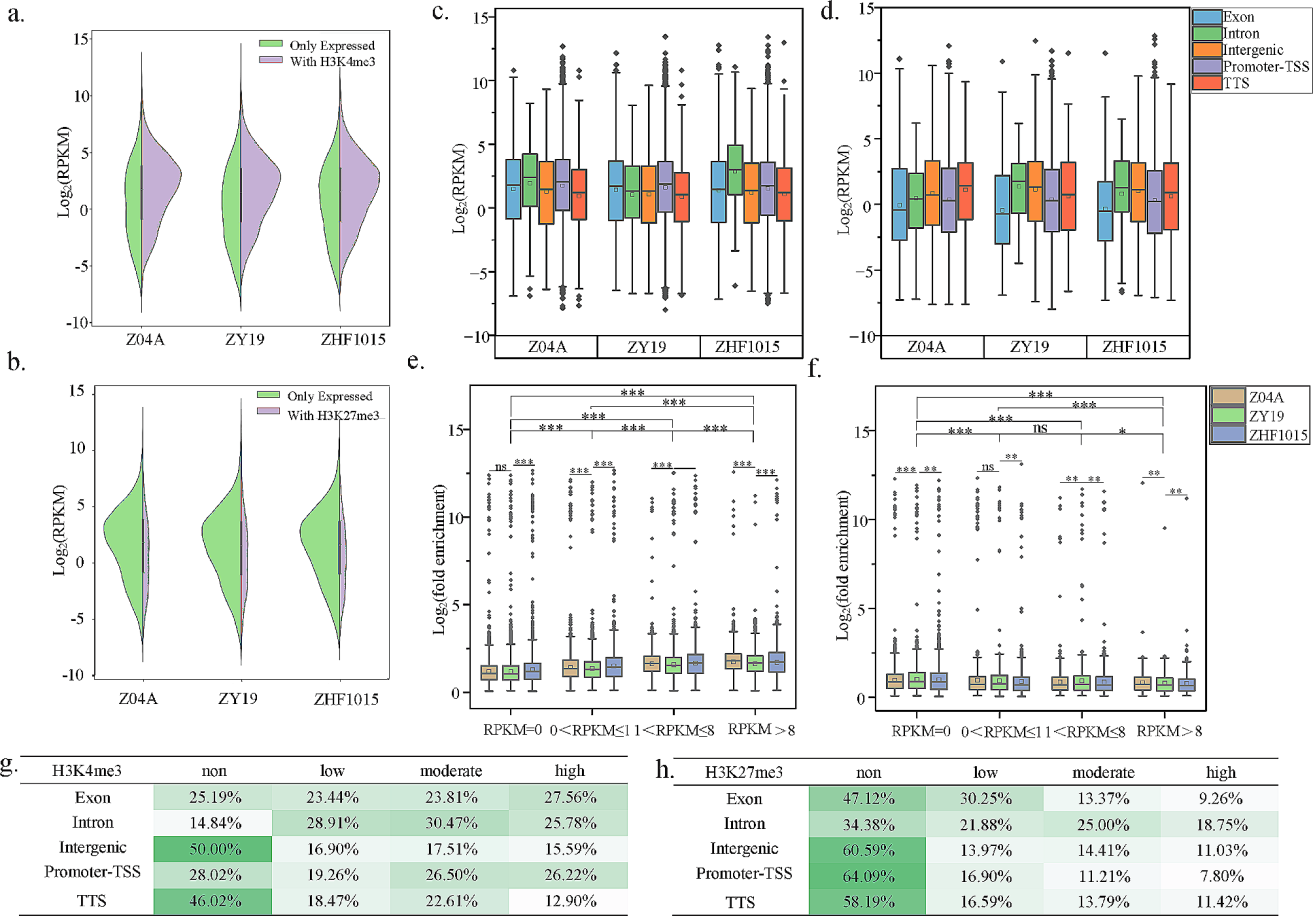
Effects of histone modifications on gene expression. (**a-b**) Expression level of genes with H3K4me3 (**a**) and H3K27me3 (**b**) modifications. (**c-d**) Expression levels of genes in different regions of H3K4me3 (**c**) and H3K27me3 (**d**) modifications. (**e-f**) H3K4me3 (**e**) and H3K27me3 (**f**) modification level of expressed genes. (**g-h**) Percentage of genes in different regions of histone modification of ZY19

Gene expression of ZY19 and its parents was calculated as RPKM (reads per kilobase genic region per million mapped reads) (Table S7). Genes targeted by H3K4me3 displayed higher expression levels in comparison to those lacking this modification (Fig. 4a). Genes targeted by H3K27me3 showed contrasting outcomes (Fig. 4b). This could be due to the fact that H3K4me3 and H3K27me3 were active and repressive markers, respectively, leading to differential effects on gene expression. The expression levels of genes within distinct histone modification regions were compared to further investigate their correlation. Genes targeted by H3K4me3 in the promoter-TSS and intron regions exhibited higher expression (Fig. 4c). Since the H3K4me3 targeted minimally in intron regions of the gene (Fig. S1d), the active effects of the marker on gene expression were mainly located in promoter-TSS regions. The repressive impacts of H3K27me3 were primarily observed in exon and promoter-TSS regions, resulting in decreased expression of the targeted genes within these areas (Fig. 4d).

To gain insight into the correlation between histone modifications and gene expression levels, expressed genes were categorized into non-expressed genes (RPKM = 0), low-expressed genes (0 < RPKM ≤ 1), medium-expressed genes (1 < RPKM ≤ 8), and high-expressed genes (RPKM > 8) (Fig. S4a, b). For H3K4me3, the number of peak modifications was higher on medium- and high-expressed genes than on low-expressed genes except for non-expressed genes (Fig. S4a). The level of modification also rose with elevated gene expression (Fig. 4e). For H3K27me3, the peak had a higher number of non-expressed genes and low-expressed genes (Fig. S4b). The modification level of non-expressed genes was significantly higher than that of expressed genes. Moreover, the modification level of the low- and medium-expressed genes was significantly higher than that of the high-expressed (Fig. 4f). Furthermore, the H3K4me3 modification levels in the hybrid consistently remained lower than those in the parents for genes exhibiting equivalent expression levels. This observation suggested that H3K4me3 modification more effectively enhances gene expression in the hybrid. Upon further examination of the regions affected by the two modifications, H3K4me3 showed a greater prevalence of medium- and high-expressed genes in exon and promoter-TSS regions compared to low-expressed genes (Fig. 4g, Fig. S4c, d). The number of H3K27me3 modifications in exon, promoter-TSS, and TTS regions decreased with elevated levels of gene expression (Fig. 4h, Fig. S4e, f).

### Combined consequences of DNA methylation and histone modification on gene expression

**Fig. 5:**
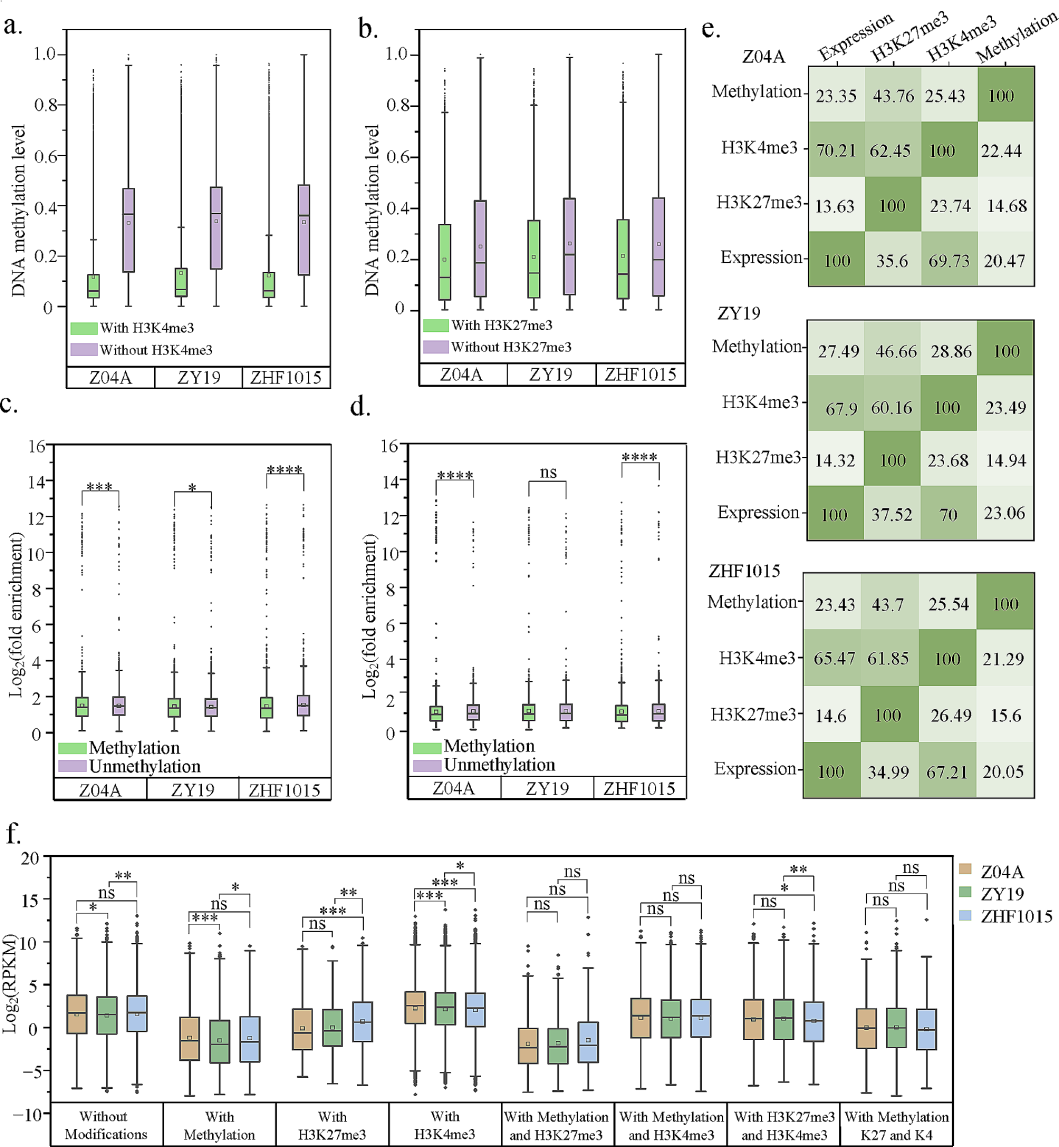
Combined consequences of epigenetic modifications on gene expression. (**a-b**) DNA methylation level of genes with histone modification. (**c-d)** Histone modification level of genes with DNA methylation. (**e**) Mutual occupancy of expressed genes, H3K27me3 target genes, H3K4me3 target genes, and DNA methylated genes. (**f**) Expression level of genes with different epigenetic modifications

Genes were modified by multiple epigenetic modifications and they often have different effects on gene expression. The DNA methylation datasets of ZY19 and its parental lines [19] were used in this study. DNA methylation genes with H3K4me3 and H3K27me3 modifications had significantly lower levels than those without. DNA methylation was more prevalent in genes without histone modification, with H3K4me3 exhibiting a stronger presence than H3K27me3 (Fig. 5a, b). Conversely, the histone modification level of genes with DNA methylation was lower than those without. Both histone modifications were more common in unmethylated genes, with this trend being more noticeable in parental lines compared to the hybrid (Fig. 5c, d). Histone modification target genes were classified by DNA methylation level to investigate their profile. Methylation levels were categorized according to previous studies, genes were divided into unmethylated genes (0 ≤ methylation level < 0.1), low methylated genes (0.1 ≤ methylation level < 0.4), moderately methylated genes (0.4 ≤ methylation level ≤ 0.6), high methylated genes (0.6 < methylation level ≤ 0.9), and fully methylated genes (0.9 < methylation level ≤ 1) [19]. Among histone modification target genes, the largest proportion was unmethylated, more than 70% in H3K4me3 and 50% in H3K27me3 (Table 1). In the hybrid, methylation was greater among histone modification target genes than in the parents. This observation could explain the lesser difference in histone modification levels between genes with and without methylation in the hybrid (Fig. 5c, d). To further understand the relationship between histone modification and DNA methylation, the number of DHM genes with DNA methylation was counted (Table 2). In the comparison of parental lines, it was observed that highly modified H3K4me3_DHM genes exhibited less DNA methylation contrary to H3K27me3_DHM genes. However, this quantitative trend was not entirely consistent in DHM_HP_s because histone modification target genes showed a higher frequency of DNA methylation in hybrid.

**Table 1:** The number of histone modification target genes with different levels of DNA methylation

Methylation level	H3K4me3 target gene (%)				H3K27me3 target gene (%)		
	Z04A	ZY19	ZHF1015		Z04A	ZY19	ZHF1015
Unmethylated	20,389 (74.57%)	19,143 (71.16%)	19,235 (74.32%)		5,558 (55.78%)	5,382 (52.59%)	6,004 (55.92%)
Low methylated	5,301 (19.39%)	5,693 (21.16%)	4,955 (19.14%)		3,007 (30.18%)	3,250 (31.76%)	3,083 (28.72%)
Moderately methylated	1,276 (4.67%)	1,511 (5.62%)	1,207 (4.66%)		1,079 (10.83%)	1,237 (12.09%)	1,223 (11.39%)
High methylated	364 (1.33%)	546 (2.03%)	470 (1.82%)		317 (3.18%)	358 (3.50%)	417 (3.88%)
Fully methylated	12 (0.04%)	8 (0.03%)	15 (0.06%)		3 (0.03%)	6 (0.06%)	9 (0.08%)

**Table 2:** The number of DHM genes with DNA methylation

Modification	Group	Z04A	ZY19	ZHF1015
H3K4me3	DHM_PP_s (M > P)	1,884	-	2,010
	DHM_PP_s (M < P)	983	-	924
	DHM_HP_s (H > M)	518	583	495
	DHM_HP_s (H < M)	419	474	361
	DHM_HP_s (H > P)	1,504	1,818	1,654
	DHM_HP_s (H < P)	418	489	412
H3K27me3	DHM_PP_s (M > P)	841	-	713
	DHM_PP_s (M < P)	703	-	730
	DHM_HP_s (H > M)	239	288	265
	DHM_HP_s (H < M)	178	172	119
	DHM_HP_s (H > P)	409	438	328
	DHM_HP_s (H < P)	131	173	165

Overlap gene counts were performed for expression, H3K27me3 target, H3K4me3 target, and DNA methylation genes. In general, the expression and H3K4me3 target genes showed higher duplication rates, amounting to approximately 70% overlap between them. In expression and DNA methylation genes, the proportion of H3K27me3 target genes was relatively low, accounting for only about 15% (Fig. 5e). In the hybrid, a higher percentage of DNA methylation genes was observed in expression, H3K27me3 target and H3K4me3 target genes compared to the parental lines, potentially indicating enhanced DNA methylation frequency. Additionally, the proportion of expressed genes was observed to be higher in the H3K27me3 target, H3K4me3 target, and DNA methylation genes compared to the parental lines (Fig. 5e).

The expressed genes were classified based on their epigenetic modifications. Genes exhibiting DNA methylation and H3K27me3 modification showed diminished expression, whereas those with H3K4me3 modification displayed elevated levels. Genes exhibiting both DNA methylation and H3K27me3 modification had the lowest expression (Fig. 5f). This implies that in cases where multiple epigenetic modifications coexist within the same gene, they might operate together and exert distinct influences on gene expression.

### Epigenetic variations of DEGs among the hybrid and parental lines

**Fig. 6:**
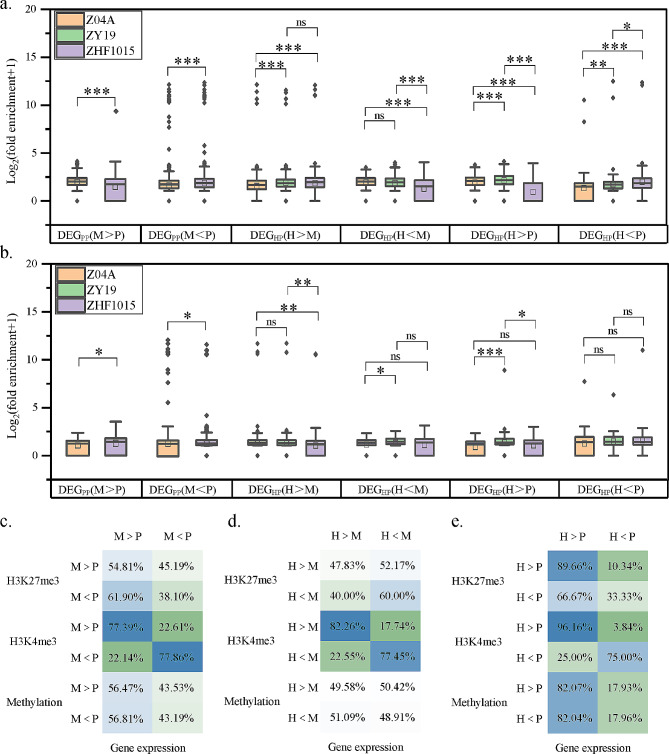
The relationship between epigenetic variation and gene expression. (**a-b**) H3K4me3 and H3K27me3 modification levels of DEGs in three cultivars. DEG_PP_ denoted DEG between the parental lines, and DEG_HP_ denoted DEG between the hybrid and its parents. H, M, and P denoted the hybrid ZY19, the maternal line Z04A, and the paternal line ZHF1015, respectively. (**c-e**) Reciprocal ratios of expression and epigenetic variation genes

The differentially expressed genes (DEG) were identified with FDR (FDR < 0.05) and fold change (|log_2_FC| ≥ 1) (Table S8). DEGs between hybrid and parents contributed to heterosis [43]. The histone modification levels of DEGs were analyzed to explore the role of their connection. For H3K4me3 modifications, higher levels of histone modifications between parental lines were always on subspecies with high expression genes. However, the pattern did not fit perfectly in DEGs between hybrid and parental lines. In the DEG_HP_(H < M) comparison between the hybrid and the maternal line, the level of H3K4me3 modification was not significantly different (Fig. 6a). For the H3K27me3 modifications, higher levels of histone modifications between parental lines were always on subspecies with low expression genes. Similarly, the pattern was not consistent in DEGs between hybrid and parental lines (Fig. 6b). The pattern of histone modifications affecting gene expression was consistent with the previous conclusions between the parental subspecies, due to the mixing of genomes, this association may become less pronounced or unstable in the hybrid.

Differences in DNA methylation and histone modifications of DEGs were statistically analyzed, revealing that only differences in H3K4me3 modifications were consistent with gene expression differences (Fig. 6c, d, e). In the comparison of the two parental subspecies, 77.39% of the gene expression and H3K4me3 modification levels were higher for Z04A than for ZHF1015, and 77.86% were lower for Z04A than for ZHF1015 (Fig. 6c). The consistency between H3K4me3 modification and gene expression was more frequent in comparisons of the hybrid with the paternal line than with the maternal line (Fig. 6d, e). The DEGs between the hybrid and the paternal line were more positively affected by the H3K4me3 modification.

**Fig. 7:**
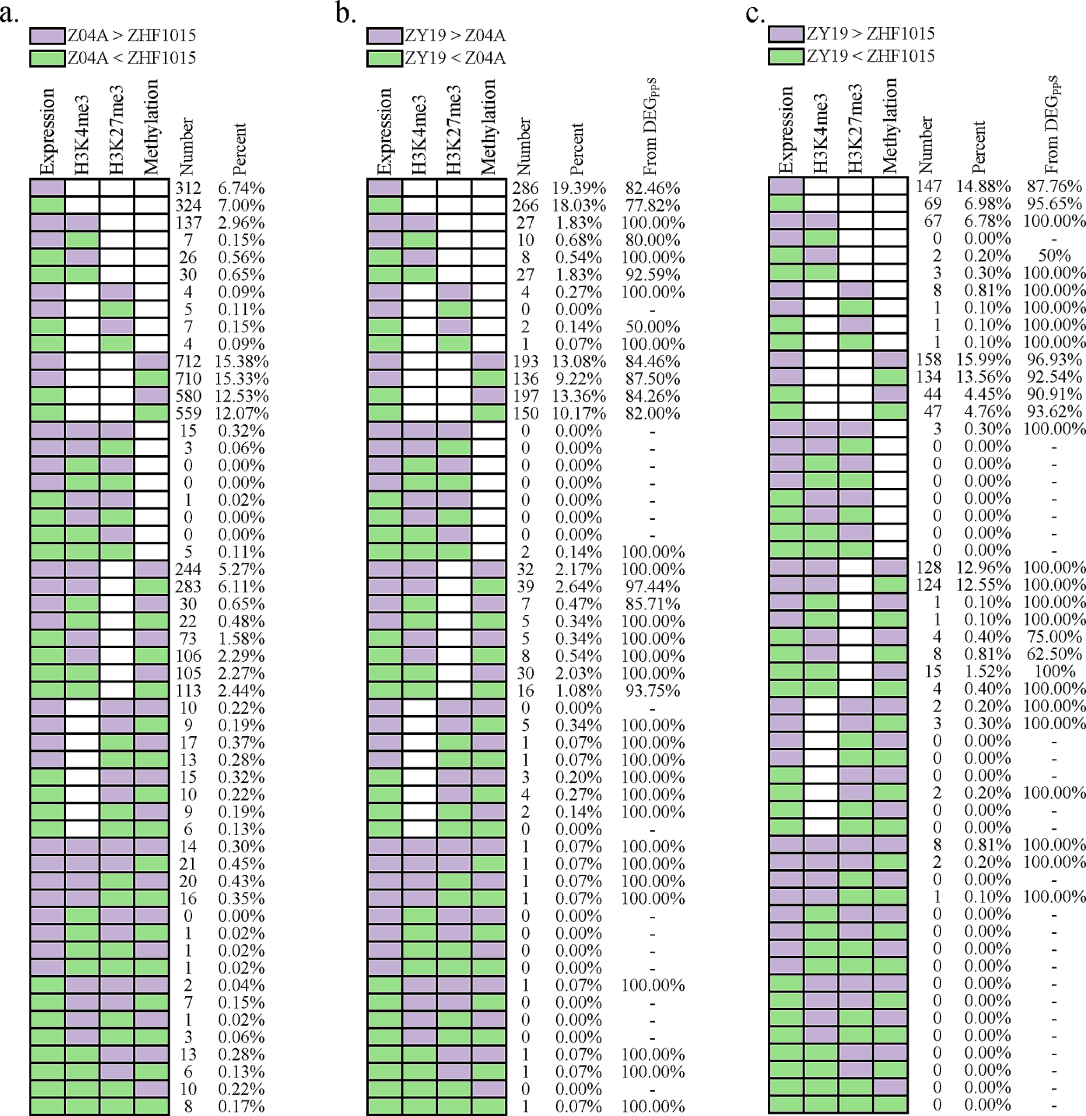
Classification of epigenetic variation in DEGs. (**a**) Number and percentage of differential epigenetic modifications in DEG_PP_. (**b-c**) Number and percentage of differential epigenetic modifications in DEG_HP_ and their percentage in DEG_PP_

To further explain the regulation of gene expression by epigenetic variations, the types and directions of epigenetic variations of DEGs were counted (Fig. 7a, b, c). Over 62.58% of DEGs were affected by epigenetic variations. In the comparison of parental subspecies, fewer DEGs had no epigenetic variations (6.74% and 7.00%). DEGs exhibiting one epigenetic modification were more positively affected by H3K4me3. DEGs exhibiting multiple epigenetic variations may be preferentially regulated by H3K4me3. When DEGs exhibited DNA methylation and H3K27me3 modification, few genes had epigenetic variations consistent with gene expression. DNA methylation and H3K27me3 repressively affected gene expression, respectively (Fig. 7a). The regulation of DEG_PP_s by epigenetic variations is equally applicable to DEG_HP_s. In addition, DEG_HP_s inherited from DEG_PP_s were counted. DEG_HP_s were more often inherited from DEG_PP_s when the H3K4me3 modification was consistent with gene expression. When DEGs were regulated by multiple epigenetic variations, most genes had H3K4me3 consistent with gene expression. Notably, the more types of epigenetic variations, the more DEG_HP_s were inherited from DEG_PP_s (Fig. 7b, c). In conclusion, the positive regulation of gene expression by the H3K4me3 variation was significant. The more epigenetic variations that lead to the formation of DEGs between parental lines, the more stable the differences in gene expression between hybrid and parental lines.

In the differential expression analysis of the hybrid in comparison to the maternal and paternal lines, 923 and 826 genes, respectively, were identified as epigenetically regulated (Fig. 7b, c). Gene Ontology (GO) enrichment analysis was conducted on these genes to elucidate their biological significance (Table S9). The comparison between the hybrid and parental lines revealed that the differentially expressed genes (DEGs) under epigenetic regulation were significantly enriched in various biological processes, including cell death, apoptosis, stress response, and phosphorylation. Additionally, these DEGs demonstrated significant enrichment in molecular functions such as ATP binding, adenyl nucleotide binding, and purine nucleotide binding. These findings suggested that these genes played critical roles in cellular stress responses and regulatory processes involving phosphorylation and kinase activities. The crucial role of epigenetic regulation in these genes highlighted their potential impact on cellular function and adaptation.

## Discussion

In hybrid and parental lines, phenotypic variation is derived from genetic and epigenetic variation [30, 44]. In comparison to their parental lines, numerous studies have demonstrated that hybrids underwent transcriptome reprogramming and epigenome remodeling in their genomes [25, 45]. In rice, studies have been made on the transcriptome and epigenetic variation mechanisms [19, 29, 31]. The effects of DNA methylation variants on rice hybrids have been found more [19, 46–48]. However, the biological significance, mode of inheritance, and regulatory mechanisms of histone modifications on gene expression are still imperfect [29, 31]. Studies on histone modifications in rice focused on the leaf or root tissues at the seedling stage [29, 31]. In this study, gene expression and histone modification regulation were investigated using the flag leaf tissues of the inter-subspecific hybrid rice ZY19 and its parents Z04A and ZHF1015 as materials. Jointly with DNA methylation [19], the relationship between changes in gene expression and epigenetic differences in the hybrids was analyzed.

### Allele-level histone modifications remodeling in inter-subspecific hybrid rice

Allele-specific epitope modification was first discovered on genomic imprinted genes [49, 50]. The phenomenon of unequal modification between alleles was found in *indica-japonica* hybrid combinations (Nipponbare and 93 − 11) [29, 51]. The generalized phenomenon was also found in both *indica-indica* hybrid rice (GL × 93 − 11 and GL × TQ), the most reported available [29]. Allele-specific histone modification genes for H3K4me3 and H3K27me3 were also identified, comprising 7.9% and 11.9%, respectively (Fig. 3a), in the inter-subspecific hybrid rice ZY19.

Differences in allelic modifications in hybrids may be due to differential modifications between the parents, or remodeling of epigenetic modifications during development [52, 53]. In the present study, 8.4% and 32.1% of DHM_PP_ genes still maintained allelic modification differences in hybrid (Fig. 3b), much less than the study by Guo et al. [29]. More genes (91.6% and 67.9%) in the *indica-japonica* hybrid underwent histone modification remodeling in the hybrid than in *indica-indica* hybrids [29], losing the histone modification differences among the parents. However, compared to the probability in allele-specific histone modifications (8.4% > 7.9%, 32.1% > 11.9%), it still can indicate that the histone modification differences between parents have a direct effect on the epigenomic composition of the hybrid. Additional analysis of the ASHM genes with distinct histone modifications in parental lines revealed 109 genes exhibiting H3K4me3_DHM_PP_ and 17 genes with H3K27me3_DHM_PP_ in hybrid, constituting 22.9% and 20.2% of all ASHM genes, respectively (Fig. 3a, b). While most of the ASHM genes (77.1% and 79.8%) were not DHM_PP_ genes, formed by remodeling in hybridization. Accordingly, it was concluded that histone modification remodeling in hybrid was the main reason for the ASHM.

In the analysis of differential histone modifications between hybrid and parents, it was found that ASHM genes had a greater probability of occurring in DHM_HP_s than in non-DHM_HP_s (Fig. 3d), which is consistent with previous studies [29]. In our study, ASHM genes in DHM_HP_s were found to be less than those reported in the study by Guo et al. Therefore, it was believed that although ASHM contributed to the formation of hybrid differential histone modifications, it was also attributable to the fact that most DHM_HP_s originated from DHM_PP_s.

No significant correlation with allele-specific expression (ASE) was found in allele-level of H3K4me3 and H3K27me3 modifications (Fig. S3a). At the allele level, H3K27me3 modification was consistent with Guo et al. and H3K4me3 modification was inconsistent with Lv et al. [29, 31]. However, when comparing the consistency of ASHM bias with ASE bias, it was found that there were more consistent than inconsistent genes in both H3K4me3 and H3K27me3 modifications (Fig. S3b). It was concluded that ASHM in hybrid contributed to ASE formation, although it did not directly regulate ASE.

### Epigenetic variations regulate gene expression in inter-subspecific hybrid rice

Epigenetic modifications had an impact on gene expression [54]. Differential epigenetic modifications between hybrid and parents affected the transcript levels of hybrid genes [19, 32, 55]. Gene expression and epigenetic modifications in hybrid and its parents were analyzed. The negative correlation of DNA methylation (Fig. 5f), the negative correlation of H3K27me3 modification (Figs. 4b, d and h and 5f), and the positive correlation of H3K4me3 modification (Figs. 4a, c and g and 5f) with gene expression were further demonstrated, in agreement with previous findings [19, 30, 32].

The frequency count of DEGs occurring simultaneously with epigenetic variations showed that only H3K4me3 modification exhibits a high degree of consistency with gene expression (Fig. 6c, d,e), with no pattern of DNA methylation and H3K27me3 as described by He et al. [51]. To explain this result, for the first time, all DEGs and epigenetic variations were systematically categorized in a differential orientation (Fig. 7a, b,c). The statistical results were interpreted. First, regarding H3K27me3 or DNA methylation variations alone regulating DEGs, their concordance pattern with DEGs was originally not significant when not influenced by other epigenetic modifications. Second, the strong influence of H3K4me3 variation on DEGs caused expression to be preferentially regulated by H3K4me3 modification when other epigenetics were present.

Only 13.74% of the DEGs had no epigenetic variations in the comparison of parental lines (Fig. 7a), and such DEGs accounted for 37.42% and 21.86% in the comparison of hybrid with maternal and paternal lines, respectively (Fig. 7b, c). It was concluded that a greater contribution of differential epigenetic modifications to DEGs was revealed in the comparison of parental lines, highlighting the significant impact of epigenetic variations on gene expression [19, 30, 32]. However, a majority of the DEG_HP_s originated from DEG_PP_s. During this process, epigenetic modifications of the hybrid were remodeled, diminishing the distinctions present between the parental lines, consequently leading to more DEGs in the hybrid with no discernible epigenetic variations.

### The effect of epigenetic modifications on heterosis in inter-subspecific hybrid rice

The formation of heterosis is underpinned by genetic differences [56]. Additionally, epigenetic modifications play a crucial role in gene expression and various biological processes [57–59]. Recent studies have further demonstrated the involvement of epigenetic modifications in regulating heterosis formation [19, 32, 60]. It is similarly evident that epigenetic variations contributed significantly to the manifestation of heterosis [51, 61, 62].

In the results of allele-level histone modification, more genes underwent histone modification remodeling in the hybrid. The occurrence of remodeling will have a series of subsequent effects that ultimately affect the phenotype of the hybrid, which may contribute to heterosis (Fig. 3a, b). Parental modification differences directly impacted hybrid epigenetic remodeling, clarifying the mechanism of heterosis from epigenetic disparities [29]. Studies have shown that epigenetic alleles can provide a genetic basis for heterosis [63]. For example, the gene *programmed cell death 5* (*OsPDCD5*, LOC_Os05g47446) played a crucial role in rice at the allelic level, involving the remodeling of histone modification. This gene was associated with photoperiod-sensitive male sterility in rice and was involved in rice programmed cell death, negatively regulating plant architecture and grain yield in rice [64]. It was observed that the H3K4me3 modification level of *OsPDCD5* in the maternal line Z04A was significantly higher than that in the paternal line ZHF1015, whereas paternal line alleles with higher modification levels in hybrid. This allele-level remodeling of histone modification of *OsPDCD5* may provide a potential candidate gene for heterosis in inter-subspecific hybrid rice. Here, allele-specific and different histone modifications were jointly analyzed, and the regulation of heterosis by DHM affecting ASHM but not depending on expression was uncovered together (Fig. 3). The conclusion that epigenetic differences between parents can directly or indirectly influence heterosis in hybrid independently of genetic differences was made early in the study of Lauss et al. [55].

The close association of differential epigenetic modification genes with DEGs was revealed [30, 51], and DEGs were critical for heterosis [43, 47, 65]. Here, DEGs were categorized with epigenetic variations, revealing the role of differences in epigenetic modifications on gene expression. Furthermore, differential modification genes had also been suggested as the epigenetic basis for heterosis [51]. The heterosis genes closely associated with epigenetic modifications have garnered significant attention in research. An example is the *Atypical S-Receptor-Like Kinase* (*OsSRK1*, LOC_Os06g13320), which regulates leaf width by promoting leaf primordial cell division. The expression level of *OsSRK1* in the hybrid was higher than that of its paternal line, and its H3K4me3 modification level was higher than that of its paternal line, which probably plays a crucial role in enhancing abscisic acid sensitivity and salt tolerance [66]. Another significant player is the *receptor for activated C kinase 1* (*OsRACK1A*, LOC_Os01g49290), which negatively regulates salt tolerance in rice [67]. The expression level of *OsRACK1A* was lower and its H3K4me3 modification level was higher than that of the maternal line in the hybrid, which may positively regulate rice seed germination by modulating endogenous ABA and H_2_O_2_ levels [68]. Both of these DEG_HP_s under epigenetic regulation serve as pivotal entities in regulating heterosis in inter-subspecific hybrid rice. Remarkably, when H3K4me3 modifications aligned with gene expression changes or when hybrid exhibited increased epigenetic variations simultaneously, DEG_HP_s were predominantly derived from DEG_PP_s (Fig. 7b, c). This may indicate that differences in epigenetic and expression between parents may be inherited synchronously by the hybrid, resulting in the more epigenetic variations that form DEGs between parents, the differences in gene expression more stable between the hybrid and parents. The H3K4me3 modification was most obvious in this process.

Some genes related to rice productivity (e.g. *GS5*, Os05g0158500; *GSN1*, Os05g0115800; *Ghd7*, Os07g0261200; *DEP1*, Os09g0441900; *An-1*, Os04g0350700; *GNP1*, Os03g0856700; *NOG1*, Os01g0752200; *LF1*, Os03g0109400) and genes associated with abiotic stress in rice (e.g. *SIT1*, Os02g0640500; *TOGR1*, Os03g0669000; *CTB4a*, Os04g0132500; *bZIP73*, Os09g0474000; *HANT*, Os11g0483000; *AETI*, Os05g0535500; *CALI*, Os02g0629800; *OsCd1*, Os03g0114800; *SNACI*, Os03g0815100) were screened for discussing allelic-specific expression and the significance of epigenetic modifications [69]. Among these genes, *TOGR1* and *SNACI* were identified as allele-specific expression genes (ASEGs) by SNPs, biased towards the maternal and paternal alleles, respectively, with roles in heat and salt tolerance. *TOGR1* exhibited lower expression levels compared to the parental levels, while *SNACI* showed higher expression levels. Regarding histone modifications, most of the screened genes were not marked with H3K27me3. Notably, *SIT1* was only modified by H3K27me3 in the hybrid, while its expression level was also higher than in the maternal line. Hybrid-specific *SIT1* modification may contribute to salt tolerance advantages. Most genes were marked with H3K4me3, an activating histone modification promoting the expression of beneficial genes. Extensive DNA methylation occurred between the hybrid and parents; similarly, most screened beneficial genes were methylated. *Ghd7* exhibited the lowest methylation levels in the hybrid, possibly explaining its higher expression levels compared to the parents. Most of these beneficial genes were expressed across three varieties, with *SNACI*, *LF1*, and *Ghd7* showing higher expression levels in the hybrid, potentially contributing to heterosis formation.

In summary, two effects of epigenetic modifications on heterosis were elucidated. First, parental differences in epigenetic modifications act on histone modification remodeling by affecting the hybrid’s ASHM, which in turn promotes heterosis. Second, epigenetic variations act on heterosis by affecting DEGs. In short, differences in epigenetic modifications and gene expression act individually or cooperatively on heterosis, providing additional evidence for epigenetic mechanisms of heterosis.

## Conclusion

In this study, genome-wide profiles of H3K4me3 and H3K27me3 modifications were analyzed in the inter-subspecific hybrid rice and its parents. The DHMs between the hybrid and its parents were more frequently observed as higher levels of modification in the hybrid than in the parents. The joint analysis of ASHM and DHM fully demonstrated the remodeling of histone modifications at the allelic level. Moreover, combined with the transcriptome and DNA methylation data, the classification of DEGs by epigenetic modification variations revealed the regulation of gene expression in inter-subspecific hybrid rice. The formation of ASHM and changes in gene expression induced by epigenetic modifications were closely associated with heterosis. In conclusion, epigenetic variations could significantly influence flag leaf gene expression in inter-subspecific hybrid rice, suggesting the identification of molecular mechanisms associated with heterosis.

### Electronic supplementary material

Below is the link to the electronic supplementary material.

Supplementary Material 1

Supplementary Material 2

Supplementary Material 3

Supplementary Material 4

Supplementary Material 5

## Data Availability

The data of RNA-Seq, ChIP-Seq, and DNA methylation are available in the National Center for Biotechnology Information (NCBI) Sequence Read Archive (SRA) with the accession numbers SRR28892180-SRR28892188, SRR28902820-SRR28902831, and SRR14935314-SRR14935321, respectively. All data analyzed during this study were included in this article and supplementary materials.

## Abbreviations

AHM: Allele-Level Histone Modification
ASE: Allele-Specific Expression
ASHM: Allele-Specific Histone Modification
BAHM: Bi Allelic Histone Modification
ChIP-seq: Chromatin Immuno Precipitation followed by next-generation sequencing
DEG: Differentially Expressed Gene
DHM: Differential Histone Modification
H3K27me3: Trimethylation on Histone H3 lysine 27
H3K4me3: Trimethylation on Histone H3 lysine 4
RPKM: Reads Per Kilobase per Million reads
