# Supplementary material for: Insights into heterosis from histone modifications in the flag leaf of inter-subspecific hybrid rice
Source: BMC Plant Biol. 2024 Aug 12;24:767. doi: 10.1186/s12870-024-05487-6 (PMC11318154; doi:10.1186/s12870-024-05487-6)
Supplement: Supplementary file 1 — Supplementary Material 1 [file 12870_2024_5487_MOESM1_ESM.docx]

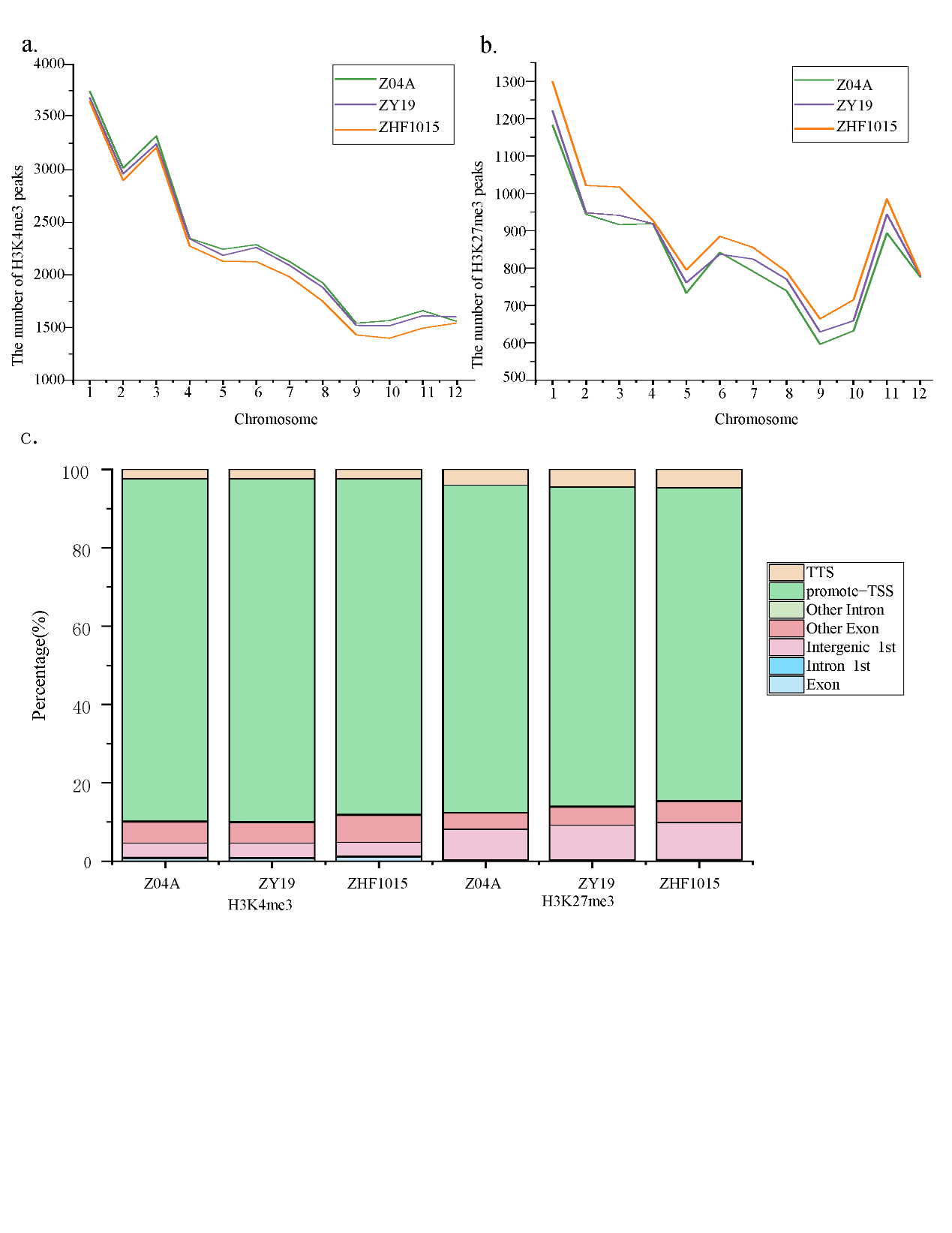


**Fig. S1** Information about histone modification peaks. The number of histone modification peaks on chromosomes (a-b), and the percentage of peaks in different regions(c).
