# Supplementary material for: Insights into heterosis from histone modifications in the flag leaf of inter-subspecific hybrid rice
Source: BMC Plant Biol. 2024 Aug 12;24:767. doi: 10.1186/s12870-024-05487-6 (PMC11318154; doi:10.1186/s12870-024-05487-6)
Supplement: Supplementary file 2 — Supplementary Material 2 [file 12870_2024_5487_MOESM2_ESM.docx]

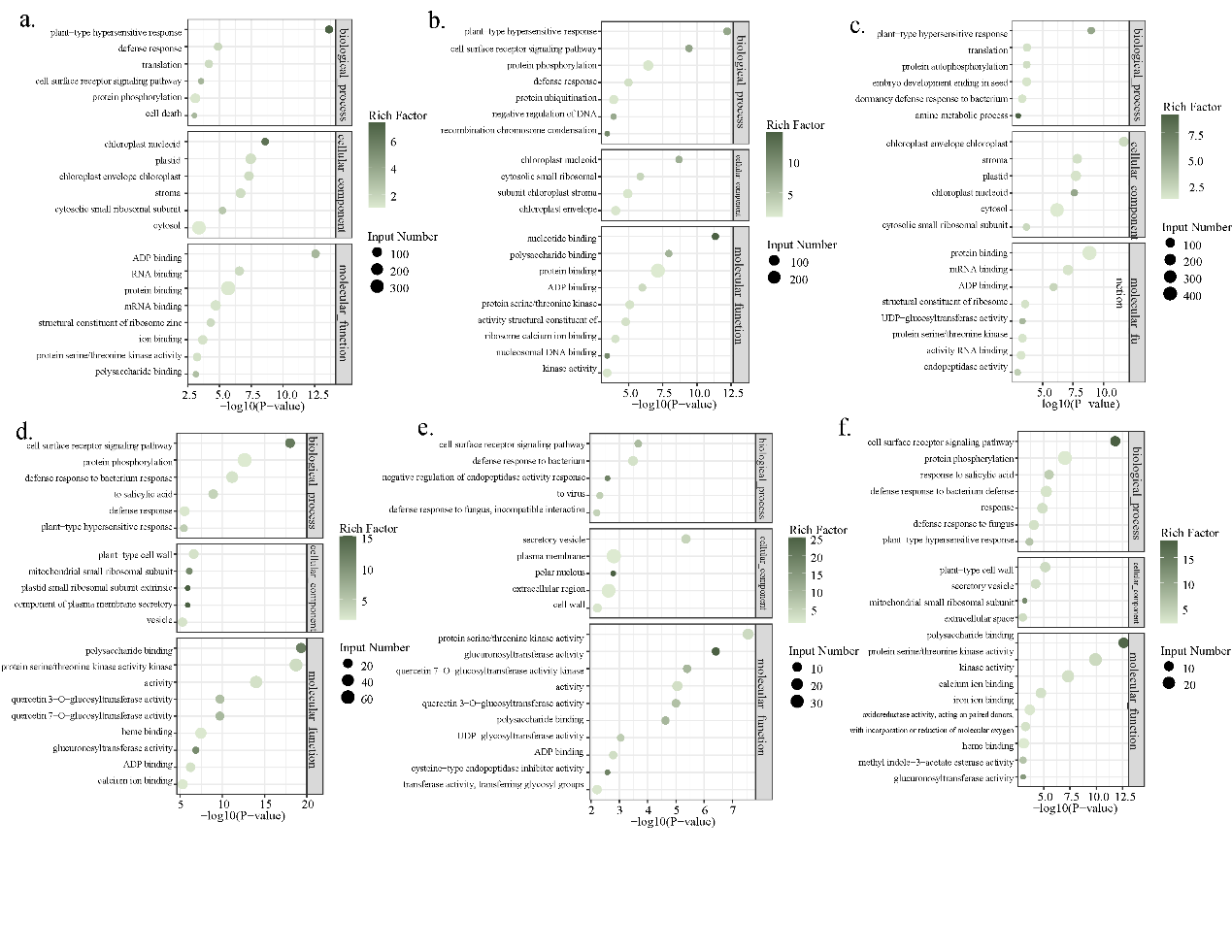


**Fig. S2** GO annotation of DHM. GO annotation of DHM_H3K4me3 genes in hybrid and its parents (a-c), and GO annotation of DHM_H3K27me3 genes in hybrid and its parents (d-f).
