# Supplementary material for: Insights into heterosis from histone modifications in the flag leaf of inter-subspecific hybrid rice
Source: BMC Plant Biol. 2024 Aug 12;24:767. doi: 10.1186/s12870-024-05487-6 (PMC11318154; doi:10.1186/s12870-024-05487-6)
Supplement: Supplementary file 3 — Supplementary Material 3 [file 12870_2024_5487_MOESM3_ESM.docx]

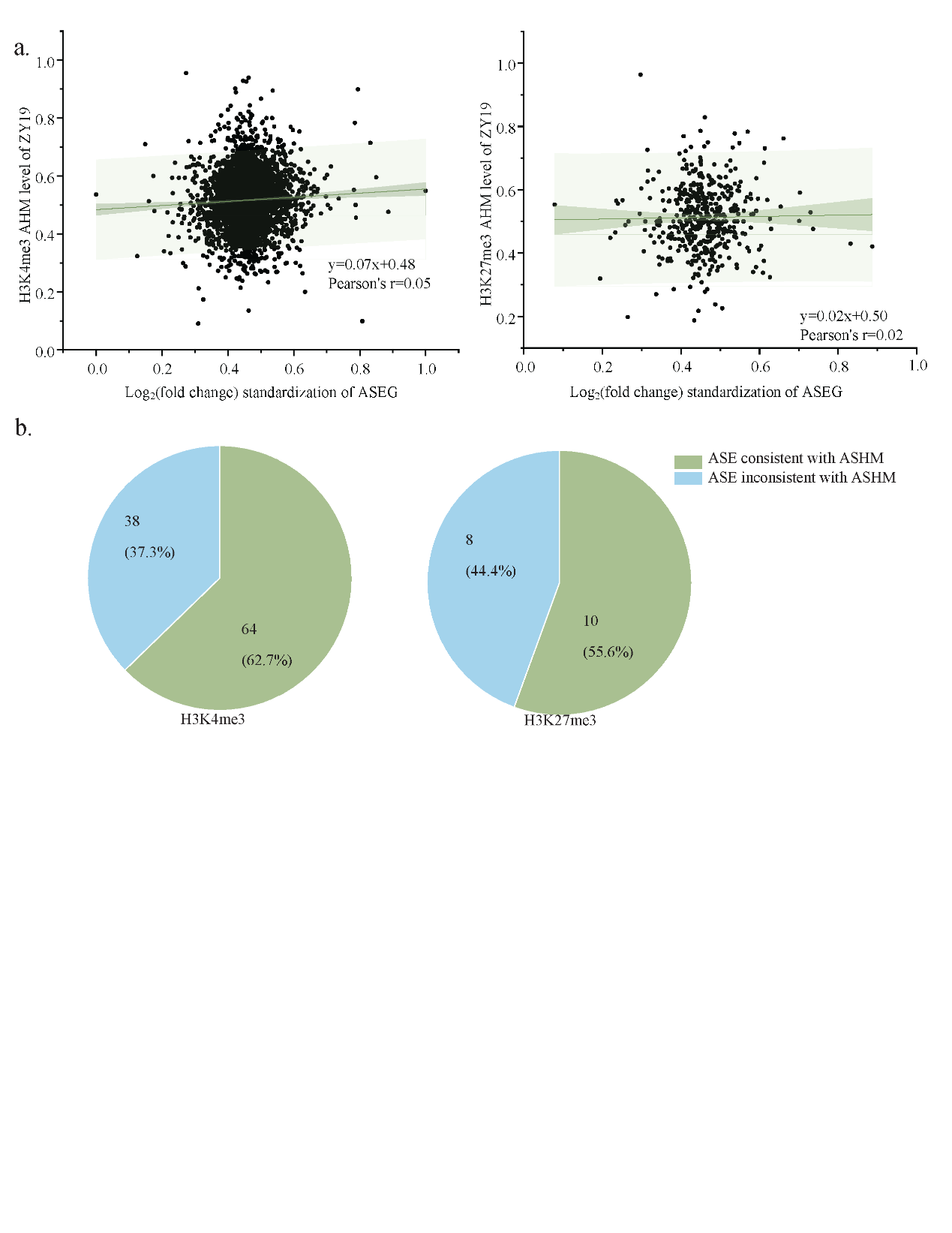


**Fig. S3** The correlation between differences in histone modifications between the parents and allele-level histone modifications (AHM) in the hybrid (a), the correlation between differences in ASE between the parents and allele-level histone modifications (AHM) in the hybrid (b), and consistency of allele-specific expression (ASE) bias and allele-specific histone modification (ASHM) bias (c).
