# Supplementary material for: Insights into heterosis from histone modifications in the flag leaf of inter-subspecific hybrid rice
Source: BMC Plant Biol. 2024 Aug 12;24:767. doi: 10.1186/s12870-024-05487-6 (PMC11318154; doi:10.1186/s12870-024-05487-6)
Supplement: Supplementary file 4 — Supplementary Material 4 [file 12870_2024_5487_MOESM4_ESM.docx]

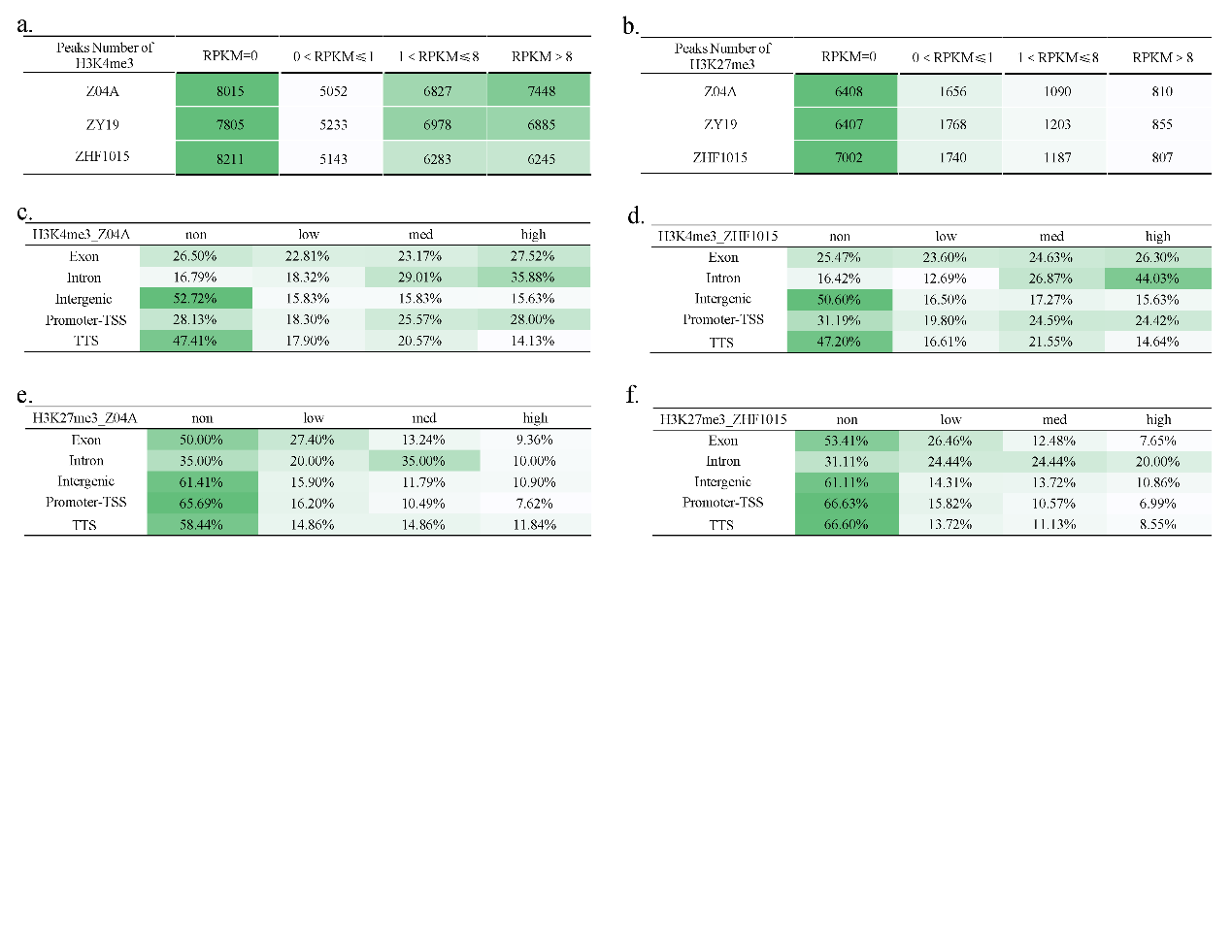


**Fig. S4** Relationship between histone modifications and gene expression. The number of histone modification peaks in different gene expression levels (a-b), percentage of gene expression in different regions of H3K4me3 modification (c-d), and percentage of gene expression in different regions of H3K27me3 modification (e-f).
